# A model linking digital media dependence, exercise empowerment, and social physique anxiety among emerging adulthood college students

**DOI:** 10.3389/fpsyg.2023.1249182

**Published:** 2023-11-24

**Authors:** Lin Luo, Junfeng Yuan, Siyuan Bi, Yanlin Wang

**Affiliations:** School of Physical Education, Guizhou Normal University, Guiyang, Guizhou, China

**Keywords:** digital media dependence, emerging adulthood, exercise empowerment, social physique anxiety, college students

## Abstract

**Background/objective:**

Social physique anxiety (SPA) is a prevalent psychological issue among emerging adults, regardless of gender. Many studies have shown that high levels of SPA are associated with various negative consequences on both physical and mental well-being. Considering the potential severity of SPA’s consequences and its high prevalence among emerging adults, it is imperative to investigate the factors and mechanisms that contribute to SPA in this population. Although prior studies have identified associations between emerging adulthood, digital media use, and SPA in young individuals, the underlying mechanisms remain unclear. The objective of this study is to examine the associations between SPA, emerging adulthood characteristics, digital media dependency, and exercise empowerment.

**Methods:**

In this cross-sectional study, Chinese college students were recruited using snowball sampling. The study utilized an online survey to assess SPA, emerging adulthood characteristics, digital media dependency, and exercise empowerment. The collected data was analyzed using path analysis.

**Results:**

A total of 1,661 Chinese college students (mean age = 19.63 ± 0.32 years, 44.97% male) were included in this study. The results showed that SPA exhibited positive correlations with responsibility and instability in emerging adulthood characteristics, digital media dependency, and exercise empowerment. Additionally, digital media dependency showed positive correlations with responsibility and instability, as well as with exercise empowerment. Furthermore, exercise empowerment demonstrated positive correlations with self-exploration, responsibility, instability, and possibilities in emerging adulthood characteristics. SPA can be directly influenced by digital media dependency, self-exploration, and instability. Furthermore, digital media dependency has a positive indirect impact on SPA through exercise empowerment. Similarly, self-exploration also has a positive indirect impact on SPA through exercise empowerment. On the other hand, instability has a negative indirect impact on SPA through exercise empowerment.

**Conclusion:**

This study provides new insights into the complex correlations with emerging adulthood characteristics, digital media dependency, exercise empowerment, and SPA. Instability, self-exploration in emerging adulthood characteristics, as well as digital media dependency, have the potential to influence SPA among college students through exercise empowerment Interventions and strategies aimed at addressing these psychological factors may prove beneficial in reducing SPA among emerging adults, especially college students.

## 1 Introduction

SPA refers to the anxiety experienced by a person when they believe their appearance is being observed or judged (Hart et al., 1989). It is considered a specific social anxiety subtype related to body image (Kowalski et al., 2006). Crawford and Eklund (1994) introduced self-presentation theory to understand the concept of SPA. In anticipated or actual social situations, individuals hope to present a positive body image to others (Ouyang et al., 2021) but doubt their ability to do so (Krane et al., 2001), leading to SPA. Numerous studies have shown that high levels of SPA are associated with harmful outcomes, including poorer mental health status (Alcaraz-Ibanez and Sicilia, 2020; Pritchard et al., 2021), disordered eating behaviors (Diehl et al., 1998), body dissatisfaction (Koyuncu et al., 2010), and increased risk of substance abuse (Roth, 2006). Emerging adulthood refers to the stage of life when an individual transitions from adolescence to adulthood, typically occurring between the ages of 18 and 29 (Arnett, 2000). During this stage, individuals experience transitions in identity exploration, education and career development, independent living, and assuming responsibilities (Reifman et al., 2007). It is a critical period for individuals to make important decisions and develop in areas such as education, career, interpersonal relationships, and self-identity (Arnett et al., 2011). SPA is a common psychological issue among emerging adults, and both men and women may experience it (Martin et al., 2006; Mülazimoğlu-Balli et al., 2010). Given the serious consequences of SPA and its high prevalence among emerging adults (Bas et al., 2004), it is urgent to understand the factors and mechanisms that affect SPA in this population.

Evidence suggests that SPA is related to digital media dependence (Harrison, 2000; Sharp et al., 2014), as well as to the motivation, attitude, preference, perceived self-efficacy, and behavior of physical activity participation (Crawford and Eklund, 1994; Sabiston et al., 2014). However, the majority of previous research on the relationships between digital media dependence, physical activity-related factors, and SPA among emerging adults has been conducted in Western societies and has primarily focused on separately examining physical activity behavior and/or digital media dependence in relation to SPA. Furthermore, there is a scarcity of research investigating the potential mechanisms that elucidate the connections between emerging adult characteristics, digital media utilization, psychological factors related to physical activity, and SPA. Therefore, there is a need for more research on SPA among emerging adults in non-Western countries. This study aims to examine the relationships between emerging adult characteristics, digital media dependence, exercise empowerment (a physical activity-related psychological factor), and SPA in a non-Western context (i.e., China) and further explore the underlying mechanisms of these relationships.

### 1.1 Emerging adulthood and SPA

Compared to other age groups such as adolescents and adults over the age of 30, emerging adults, including college students, have unique characteristics (Arnett, 2000; Reifman et al., 2007; Arnett et al., 2011). This period is characterized by significant changes in the living and social environment, including independent living or romantic relationships (Arnett, 2000; Kuang et al., 2023). The changes can be so significant that emerging adults require a considerable amount of support to successfully navigate this transition period (Reifman et al., 2007). The characteristics of emerging adulthood have been described as identity exploration, instability, self-focus, autonomy, and possibility (Arnett, 2000). Previous studies have found that emerging adulthood characteristics are related to psychological variables such as happiness (Reifman et al., 2007), risk behavior, self-esteem (Galambos and Martínez, 2007), depression (Luyckx et al., 2011), and social anxiety (Lanctot and Poulin, 2018) in emerging adults. It has been shown that emerging adults are more prone to SPA (Alcaraz-Ibáñez et al., 2020; Alcaraz-Ibanez and Sicilia, 2020; Pritchard et al., 2021; Swami et al., 2021) since body image is their primary concern (Neumark-Sztainer et al., 2018). There is also evidence that mass media’s idealization of body image can induce SPA in emerging adults (Sabiston and Chandler, 2009), and women who spend more time on social media have higher SPA (McColgan and Paradis, 2022). These findings indicate the complexity of factors that influence SPA in emerging adults.

Why do emerging adults tend to experience SPA? This may be related to the important transitional period of identity and physical development in emerging adulthood. In the process of self-exploration, emerging adults face numerous complex and constantly changing choices that encompass various aspects of their lives, including career, education, interpersonal relationships, love, values, and more. These choices can lead to instability in their lives. The characteristics of self-attention mean that young people in this life stage are more focused on themselves in their choices. This is because, compared to adolescents, they are less influenced by teachers, parents, and other elders during this period, and they also have fewer responsibilities in life. By making their own decisions, emerging adults also learn to be self-sufficient. The feeling of being in the middle means that emerging adults have already gained a certain degree of independence. Although emerging adults have gained a certain degree of autonomy, being away from their parents, they may not be ready to take on the role of an adult. Additionally, young people at this stage of life have boundless potential because they have not yet settled down, allowing them to explore various life possibilities. SPA is related to anxiety related to the body, and emerging adults are particularly concerned about their perceptions and attitudes toward their bodies (Neumark-Sztainer et al., 2018). At the same time, interpersonal characteristics during emerging adulthood often involve the development, re-evaluation, and maintenance of romantic, sexual, and peer relationships (Sabiston et al., 2014). Negative feedback on one’s appearance may lead to greater attention to one’s appearance and poorer psychological health (Cash et al., 2004). In turn, high levels of self-focus may increase dissatisfaction with one’s body image (Swami et al., 2021) and lead to the anticipation of negative evaluations from others, resulting in decreased confidence and greater social anxiety in interpersonal environments. Therefore, the study hypothesizes that the characteristics of emerging adults can significantly affect their SPA.

### 1.2 Digital media dependence and SPA

This generation, known as emerging adults, is the first to grow up in the digital world, with 90% of them using digital media such as social networking sites (Barrett, 2020). Although adolescents are frequent media users (Rideout and Inc, 2015), emerging adults who are for the first time away from parental supervision are particularly likely to increase their media use (Coyne et al., 2013). Digital media has a great influence on emerging adults, and social media plays an important role in the transformation of emerging adult characteristics (Frozzi and Mazzoni, 2011; Strickland, 2014). Studies have shown that digital media use can have positive effects in some aspects, such as increasing social support from friends (Hampton et al., 2011), self-exploration (Michikyan et al., 2015), and greater self-esteem after receiving feedback from friends (Valkenburg et al., 2006). In addition to popular culture motivations, digital media provides emerging adults with a quick way to establish interpersonal relationships to consolidate their offline identities (Bahk et al., 2010). However, some studies have also shown that digital media can lead to negative outcomes (Stice et al., 2000; Thompson and Stice, 2001). For example, mass media tends to portray the “ideal body type” of young people according to its own perspective. Emerging adults who are in this objectifying environment, influenced by the media’s perspective, are prone to self-objectification. They unconsciously accept the “indoctrination” of this environment and transform it into their own “body ideal” through an internalization process (Stice et al., 2000; Thompson and Stice, 2001). Due to the role of mass media as a source of ideal appearance information, emerging adults who are overly exposed to media images of slim female models or muscular male models will internalize the ideal esthetic standards promoted by the media and perceive them as a real and universal standard (Qin, 2009; Galioto and Crowther, 2013). At the same time, mass media is often considered an important factor in causing individual body image disorders, because the body shapes of models, celebrities, and actors conveyed by the media are difficult to achieve. When the gap between the real self-body and the ideal body produced by oneself under the influence of the media was large, it led to a decrease in self-body satisfaction (Smeets et al., 2010, 2011). Therefore, emerging adults who use digital media more are more likely to be influenced by mass media, resulting in dissatisfaction with their own body image. Digital media dependence refers to an individual’s usage rate of digital media, and the higher the usage rate, the higher the level of dependence on digital media (Smeets et al., 2011). Previous studies have shown that the stronger the media dependence, the greater the psychological pressure and negative body image, and the higher the level of SPA (Bas et al., 2004; Martin et al., 2006; Mülazimoğlu-Balli et al., 2010). Therefore, the study hypothesizes that digital media dependence can significantly affect emerging adults’ SPA.

### 1.3 The mediating role of exercise empowerment

Conger and Kanungo (1988) viewed empowerment as an inner motivation and psychological authorization experience. It is believed that empowerment is a process of enhancing self-efficacy and the necessary conditions for internal motivation. Blinde et al. (1993) described the empowerment developed by female college athletes through sports participation as “physical ability, perception of self-ability, and a proactive lifestyle.” Moore and Fry (2014, 2017) showed that individuals who believe they can positively influence their physical and mental health through sports participation, i.e., gain exercise empowerment, including benefits from their current physical education experience (e.g., improving weight, increasing health, reducing pain, and lowering blood pressure), and particularly emphasized the transfer of their exercise experience outside the classroom (Sabiston and Chandler, 2009; Moore and Fry, 2014, 2017; Sabiston et al., 2014). Perceptions of exercise empowerment may help explain why some participants can successfully overcome obstacles on the road to achieve long-term exercise behavior changes and to achieve their personal health goals. Physical activity is essentially social and evaluative, placing strong emphasis on the form and function of the body (Sharp et al., 2014). SPA arises from the influence of social anxiety, self-presentation, and body image, which can be a source of or motivation for physical activity. Crawford and Eklund (1994) further described SPA as a self-presentation anxiety related to body shape (such as body fat, muscle density, and body proportion). Given these factors, it is not surprising that SPA is related to exercise participation motivation, attitudes, preferences, and perceptions of self-efficacy (Sharp et al., 2014). Previous studies have found that exercise self-presentation factors (body tension, weight control, and physical attractiveness) can predict SPA in physically active women (Eklund and Crawford, 1994). Therefore, exercise empowerment may also be able to predict SPA.

**Figure 1:**
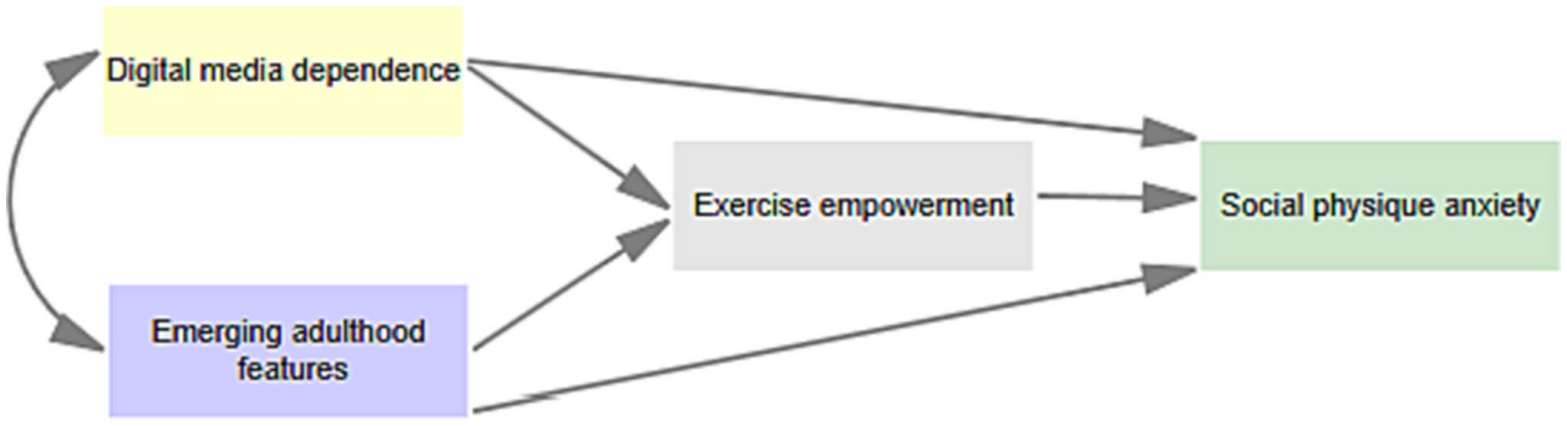
Hypothesized model.

Digital media provides the environmental factors necessary for empowering emerging adults through physical activity, which may lead to increased participation in physical activity to gain positive evaluations from others. However, this may also lead to a higher experience of SPA as their bodies are constantly evaluated. Therefore, the study hypothesizes that exercise empowerment, as an internal motivation and psychological empowerment experience in physical activity, may mediate the relationship between digital media dependence and SPA. The 1995 National College Health Risk Behavior Survey found that more than one third of students (36%) did not participate in adequate amounts of physical activity (Douglas et al., 1997). Other studies found an even higher percentage of college students (about 40–50%) who were not physically active (Gregg et al., 2002). These findings may be attributed to the instability experienced during early adulthood, especially during the period of self-exploration, which may lead to a reduction in the time spent participating in physical activity and a shift to other activities such as dating and job hunting. Such behavioral changes in a new environment may affect their participation in physical activity, which is significantly related to exercise empowerment. Previous studies have confirmed the association between emerging adulthood characteristics and physical activity levels among college students (Kuang et al., 2023). Therefore, the study hypothesizes that exercise empowerment may mediate the relationship between emerging adulthood characteristics and SPA. Given the high prevalence of digital media use among young adults, the study also hypothesizes that emerging adulthood characteristics are associated with digital media dependence. In summary, the study hypothesizes that emerging adulthood characteristics and digital media dependence are associated with exercise empowerment, which may then affect their SPA. The proposed model is shown in Figure 1.

## 2 Methods

### 2.1 Study design and participants

This study employed a cross-sectional research design. The participants were Chinese college students recruited from a university in the southwestern region of China using snowball sampling. Data was collected through an online survey, which included variables such as SPA, emerging adulthood characteristics, digital media dependency, and exercise empowerment. Path analysis was used to assess the associations between SPA and emerging adulthood characteristics, digital media dependency, and exercise empowerment.

In order to estimate the minimum sample size to fit the prevalence of SPA as closely as possible, this study adopted the formula recommended by the World Health Organization (WHO) (Lwanga et al., 1991). The formula for sample size calculation is as follows: Where *n* is the sample size; *Z* is the *Z* statistic at the 1*-α/*2 confidence level; *P* represents the expected prevalence; *d* is the precision level (i.e., allowable absolute error level); and *deff* represents the design effect factor.


$$n=\frac{Z^{2}P\left(1-P\right)}{d^{2}}\times \text{deff}$$

According to previous studies (Herring et al., 2020), the estimated prevalence rate of SPA is approximately 5.1%. However, there is currently no data available on the prevalence rate of SPA in China. Therefore, this study sets the expected rate of SPA prevalence (*P*) at 5%. Assuming a Type I error (*α*) of 0.05, Z_1-α/2_ of 1.96, an acceptable absolute error level (*d*) of 2%, and an efficiency factor (*deff*) of 2.0, the minimum required sample size (*n*) is calculated to be 1,250. Additionally, considering the need to exclude invalid or non-eligible data during the data survey, a data exclusion rate of 30% is set. Therefore, a minimum sample size of 1,185 cases is required.

The sample for this study consisted of 1,700 college students from China, with an average age of 19.63 years (standard deviation = 0.32). Participants who met the following inclusion criteria were included in the study: (i) aged between 18 and 29 (Kuang et al., 2023); (ii) no physical disabilities; (iii) able to freely use digital media tools; (iv) not diagnosed with any mental or neurological disorders. A total of 39 participants were excluded due to being outside the age range of emerging adulthood or not completing the entire survey. Ultimately, 1,661 college students from different provinces were included in the study.

### 2.2 Measurement

#### 2.2.1 Demographic information

Demographic information collected included gender (male and female), age, ethnicity (Han and minority), household registration (urban and rural), grade (freshman, sophomore, junior, senior, graduate (master), and major (humanities, science, engineering, arts).

#### 2.2.2 Emerging adulthood characteristics

Since the proposal of the five characteristics of emerging adulthood (Arnett, 2000), Reifman et al. (2007) developed a tool called the Inventory of Dimensions of Emerging Adulthood (IDEA) to measure individuals’ perceptions of emerging adulthood. During the development of the original IDEA-31, an additional dimension called “other-focused” was added, resulting in six subscales: identity exploration, experimentation/possibilities, negativity/instability, feeling in-between, self-focused, and other-focused. In this study, the Chinese version of the Inventory of Dimensions of Emerging Adulthood (IDEA-C) validated by Kuang et al. (2023) was used. IDEA-C consists of four dimensions: self-exploration (e.g., “Time of trying out new things”), instability (e.g., “Time of feeling stressed out”), possibilities (e.g., “Time of many possibilities”), and settling down (e.g., “Time of settling down”). All items were rated on a 4-point Likert scale, ranging from 1 (“strongly disagree”) to 4 (“strongly agree”), with higher scores indicating higher perceived levels. The Chinese version of IDEA showed good internal consistency (Cronbach’s alpha >0.77) and test–retest reliability (*r* > 0.49, *p* < 0.01). The Cronbach’s alpha for this study was 0.888.

#### 2.2.3 SPA

SPA was measured using the SPA scale developed by Hart et al. (1989). The scale is a unidimensional structure with 16 items, of which only item 1 (“I feel comfortable with how I look in front of others.”) is reverse-scored. Each item is rated on a 5-point Likert scale, ranging from 1 (strongly disagree) to 5 (strongly agree). The study used the Chinese version of the scale (Di Gesto et al., 2023). Higher total scores indicate higher levels of SPA. The Cronbach’s alpha of the scale was 0.970 in this study.

#### 2.2.4 Digital media dependence

The digital media dependence scale used in this study was adapted from Bahk et al. (2010). The Chinese version of the scale consists of 12 items, which form a one-dimensional scale (e.g., “I prefer sending emails to people rather than talking face-to-face”). These items were rated on a 5-point Likert scale, ranging from 1 (“strongly disagree”) to 5 (“strongly agree”). The total score was obtained by summing all the answers. A higher total score indicates a higher degree of individual digital media dependence. Analysis using the Rasch model revealed that the reliability and validity of the Chinese version of the digital media dependence scale were acceptable, with a Cronbach’s alpha coefficient of 0.930.

#### 2.2.5 Exercise empowerment

The Exercise Empowerment scale used in this study was adapted from Moore and Fry (2014). In this study, the Chinese version of the Exercise Empowerment scale was used to assess individuals’ exercise perception, efficacy, and other related factors. This is a unidimensional scale consisting of 13 items (e.g., “I believe exercise can have a positive impact on my general physical health (e.g., blood pressure, pain relief, weight management)”). These items are rated on a 5-point Likert scale ranging from 1 (“strongly disagree”) to 5 (“strongly agree”). The sum of all answers is the total score. The higher the total score, the higher the level of individual exercise empowerment. The reliability and validity of the Chinese version of the Exercise Empowerment scale in this study were acceptable, with a Cronbach’s alpha of 0.951.

### 2.3 Data analysis

Statistical analysis was performed using IBM SPSS 26.0 and IBM SPSS Amos 23.0 (IBM SPSS Statistics for Windows, NY: IBM Corp). This study used the Harman single-factor test to test for common method biases. Setting the common factor to 1, all items were used as manifest variables for confirmatory factor analysis. The results of the confirmatory factor analysis showed that the model fit indices (*χ*^2^*/df* = 30.830, GFI = 0.393, IFI = 0.354, NFI = 0.386, RMSEA = 0.134) were not ideal. This indicates that there is no significant common method bias between variables. Therefore, in further statistical analysis, no variable was excluded or merged. The normal distribution of the variables of interest, including age, emerging adulthood characteristics, digital media dependence, exercise empowerment, and SPA, was checked using the Doornik-Hansen test. The above variables all met normal distribution. Independent samples t-tests were performed to determine gender differences in age and the normally distributed variables mentioned above, while chi-square tests were used to compare gender differences in other demographic statistics. In addition, the correlation between each pair of variables mentioned above was studied using Spearman rank correlation coefficient. The rating of correlation was as follows: *r* ≥ 0.8 = large, *r* < 0.8 to >0.2 = medium, *r* ≤ 0.2 = small.

The study used the maximum likelihood (ML) estimation method, and recommended indices as follows: (1) Standardized *χ*^2^, i.e., the chi-square value divided by the degrees of freedom; (2) Comparative Fit Index (CFI); generally, a CFI value over 0.95 indicates good fit, and a value of 0.90 to 0.95 is acceptable; (3) Root Mean Square Error of Approximation (RMSEM), where a reasonable fit standard is <0.06; (4) Standardized Root Mean Square Residual (SRMR), where a value lower than 0.08 indicates a relatively good model fit. In all statistical tests, the statistical significance level was set to *α* = 0.05. Finally, using 5,000 samples and 95% confidence intervals of Bootstrapping, the total effects, indirect effects, and direct effects between variables were determined through AMOS.

## 3 Results

### 3.1 Participant characteristics

Participant characteristics are listed in Table 1.

**Table 1:** Participants’ demographics and characteristics (*N* = 1,661).

	Total (*n* = 1,661)	Men (*n* = 747)	Women (*n* = 914)	*p*-value
	*n* (%) or Mean ± SD			
Age (years)	19.63 ± 0.32	19.73 ± 0.53	19.55 ± 0.38	<0.005
*Ethnicity*				
Han	933(56.17%)	434(58.10%)	499(54.60%)	0.152
Minorities	728(43.83%)	313(41.90%)	415(45.40%)	
Household registration				0.497
Cities	276(16.62%)	119(15.93%)	157(17.18%)	
Town and rural	1,385(83.38%)	628(84.07%)	757(82.82%)	
Grade				<0.001
Freshman year	644(38.83%)	324(43.37%)	321(35.12%)	
Sophomore year	903(54.46%)	362(48.46%)	541(59.19%)	
Third Year	61(3.67%)	53(7.10%)	8(0.88%)	
Senior year	48(2.89%)	5(0.67%)	43(4.70%)	
Graduate students	4(0.24%)	3(0.40%)	1(0.11%)	
Major				<0.001
Human Sciences	463(27.87%)	114(15.26%)	349(38.18%)	
Science category	589(35.46%)	267(35.74%)	322(35.23%)	
Industrial Sciences	540(32.51%)	334(44.71%)	206(22.54%	
Arts and Sports	69(4.15%)	32(4.28%)	37(4.05%)	

### 3.2 Descriptive and correlation analyses

Table 2 shows the descriptive analysis of participants’ four characteristics of emerging adulthood: self-exploration, responsibility, instability, and possibility, as well as their levels of digital media dependence, exercise empowerment, and SPA. The correlation results showed that SPA was positively correlated with responsibility, instability, digital media dependence, and exercise empowerment. Exercise empowerment was positively correlated with self-exploration, responsibility, instability, possibility, and digital media dependence. Digital media dependence was positively correlated with responsibility and instability. The four characteristics of emerging adulthood: self-exploration, responsibility, instability, and possibility, also showed significant correlations with each other. The correlation results between each pair of variables are shown in Table 2.

**Table 2:** Descriptive and correlation analyses (*N* = 1,661).

Variables	1	2	3	4	5	6	7
1. Self-exploration	–						
2. Responsibility	0.52^**^	–					
3. Instability	0.40^**^	0.36^**^	–				
4. Possibilities	0.67^**^	0.44^**^	0.38^**^	–			
5. Digital media dependency	0.04	0.14^**^	0.22^**^	−0.01	–		
6. Exercise empowerment	0.37^**^	0.29^**^	0.09^**^	0.32^**^	0.20^**^	–	
7. SPA	−0.01	0.12^**^	0.27^**^	−0.03	0.74^**^	0.16^**^	–
Total (Mean)	24.62	11.20	14.16	9.25	37.09	49.12	35.61
Total (SD)	0.09	0.05	0.07	0.04	0.23	0.20	0.27

**p < 0.01.

### 3.3 Testing of the hypothesized path model

**Figure 2:**
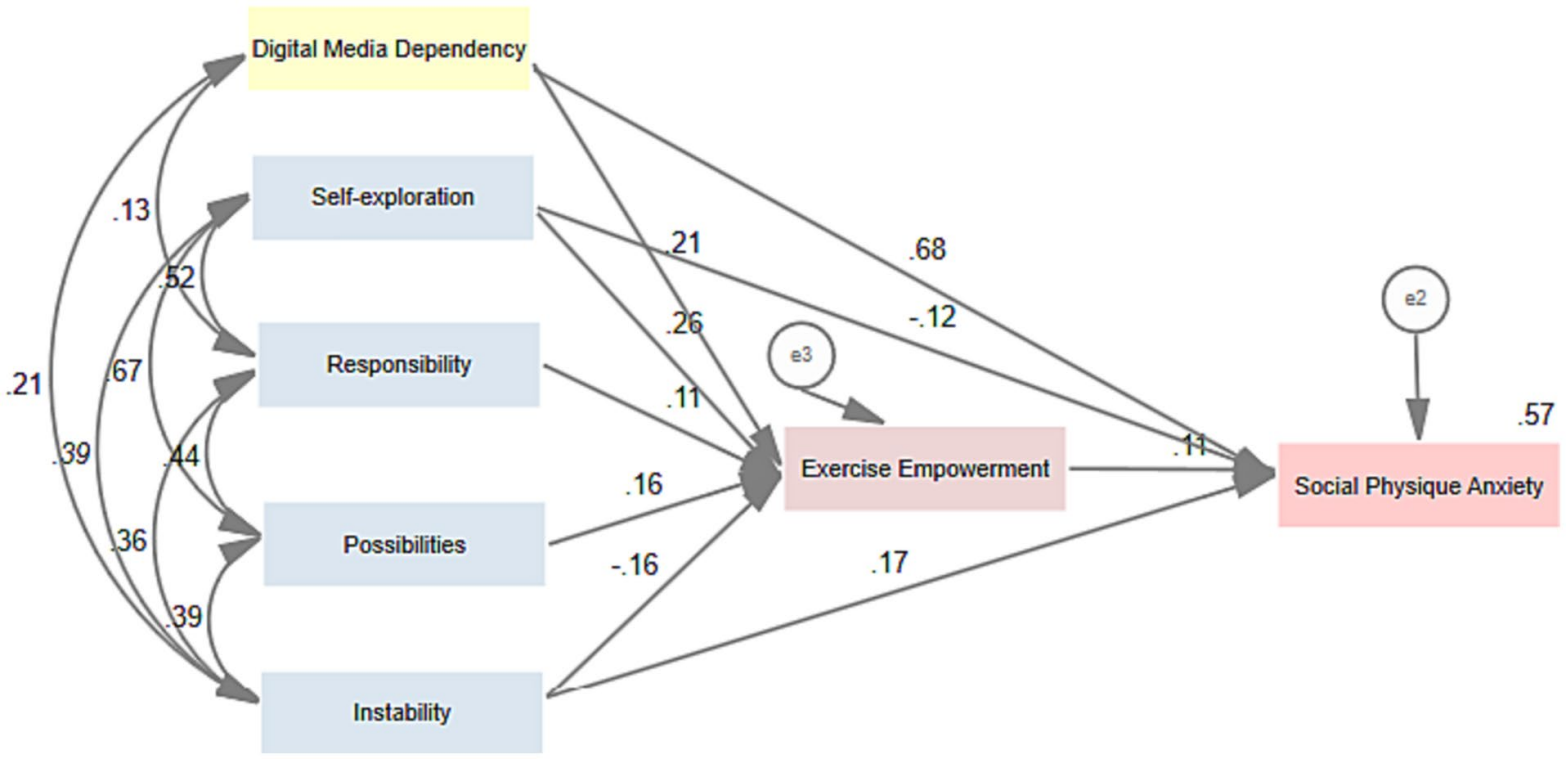
Causal path model.

The results showed that the model fit was acceptable based on the following model indices (*χ*^2^ = 11.274, *df* = 4, RMSEA = 0.033, CFI = 0.998, *p* < 0.05). In addition, the standardized regression coefficients are shown in Figure 2 and Table 3.

**Table 3:** Direct effects and indirect effects in SEM (*N* = 1,661).

Path	Direct effects			Indirect effects			Direct effects
	Coefficient*	S.E.	*p*-value	Coefficient*	S.E.	*p*-value	Coefficient*
Resp → EE	0.114	0.105	<0.001	No Path			0.114
Poss → EE	0.155	0.153	<0.001	No Path			0.155
Inst → EE	−0.158	0.078	<0.001	No Path			−0.158
Self → EE	0.261	0.074	<0.001	No Path			0.261
DMD → EE	0.206	0.020	<0.001	No Path			0.206
Self → SPA	−0.117	0.071	<0.001	0.030	0.000	<0.001	−0.087
Inst → SPA	0.167	0.090	<0.001	−0.018	0.000	<0.001	0.149
DMD → SPA	0.675	0.025	<0.001	0.024	0.000	<0.001	0.699
EE → SPA	0.114	0.029	<0.001	No Path			0.114

*Standardized coefficients; DMD = Digital Media Dependency, EE = Exercise Empowerment, SPA = Social Physique Anxiety, Self = Self-exploration, Inst = Instability, Poss = Possibilities, Resp = Responsibility.

Lines represent significant paths. The numbers on the arrows represent the standardized path coefficients.

### 3.4 Assessing direct, indirect, and total effects

First, as shown in Table 3, the results indicate that digital media dependence (*β* = 0.675, *p* < 0.01), self-exploration (*β* = −0.117, *p* < 0.01), and instability (*β* = 0.167, p < 0.01) have a direct impact on SPA. Second, digital media dependence has a positive indirect effect on SPA through exercise empowerment (*β* = 0.024, *p* < 0.01). Third, self-exploration has a positive indirect effect on SPA through exercise empowerment (*β* = 0.030, *p* < 0.01). Fourth, instability has a negative indirect effect on SPA through exercise empowerment (*β* = −0.018, *p* < 0.01). For the results of other paths, see Figure 2 and Table 3.

## 4 Discussion

This study investigated the complex relationships among emerging adult characteristics, digital media dependence, exercise empowerment, and SPA. Overall, our results supported our theoretical model and emphasized the importance of emerging adult characteristics, digital media dependence, and exercise empowerment for SPA. Specifically, digital media dependence was positively correlated with SPA and exercise empowerment. Digital media dependence could directly affect SPA and indirectly affect SPA through exercise empowerment. In particular, college students who reported higher levels of self-exploration exhibited lower levels of SPA. College students who reported higher levels of instability exhibited higher levels of SPA. Exercise empowerment played a mediating role in the effects of self-exploration and instability on SPA.

This study found that the use of digital media, particularly digital media dependency, plays a significant role in the occurrence of SPA among college students. Di Gesto et al. (2023) conducted a study on 201 Italian women (average age 22) and found that participants who had been exposed to Instagram pictures with a high number of likes showed higher body dissatisfaction and SPA. Similarly, Ruiz et al. (2021) found a positive correlation between SPA and social networking site (SNS) addiction in a study of 439 teenagers. However, there are also some studies with different conclusions. McColgan and Paradis (2022) conducted a one-week longitudinal study on 214 women and found no significant relationship between overall social media usage time and SPA. Despite the disparate findings, interventions targeting the processes of digital media use may help alleviate SPA levels among college students. The media dependency theory provides a multi-level ecological framework for understanding the significance of media in individual and social daily life. Many scholars have found that digital media dependency among emerging adults predicted various individual behaviors and attitudes (Hu, 2017). Given the high prevalence of SPA among emerging adults, developing interventions or strategies to actively improve their digital media usage holds practical significance and may provide new perspectives for intervening in SPA among emerging adults. For example, researchers had effectively reduced participants’ digital media dependency and improved their psychological well-being by using mindfulness interventions to reduce their reliance on smartphones (Bahk et al., 2010). Further research is needed to explore how to reduce digital media dependency among emerging adults, which may lead to a reduction in the occurrence or severity of SPA.

This study found a positive correlation between exercise empowerment and SPA. Physical activities have social and evaluative aspects, emphasizing the form and function of the body. Exercise empowerment can enhance and develop individuals’ physical abilities through participation in various sports and physical activities (Moore and Fry, 2017). Furthermore, physical activity also has a positive impact on exercise empowerment. Physical activity is the foundation for achieving exercise empowerment, and only through active participation in various physical activities can the effects of exercise empowerment be obtained (Moore and Fry, 2014). Therefore, there is a mutually reinforcing relationship between exercise empowerment and physical activity. SPA is influenced by social anxiety, self-presentation, and body image and can be influenced by or affect physical activity behavior. Jankauskiene and Baceviciene’s (2019) study found a link between reduced physical activity and higher SPA. Herring et al.’s (2020) research report showed that individuals with low levels of physical activity had higher SPA scores, and SPA played a mediating role between physical activity and anxiety. Bartlewski’s (1996) study on aerobic exercise intervention for female college students found that regular aerobic exercise programs significantly reduced SPA in female college students. However, Niven et al. (2009) found that SPA was unrelated to current or future physical activity in their sample. In addition to being influenced by physical activity, exercise empowerment is also affected by other factors such as obesity (Morgan, 2004) and body appreciation (Nargis, 2017). Eklund and Crawford (1994) found a positive correlation between reasons for self-presentation in exercise (body tone, weight control, and physical attractiveness) and SPA. Therefore, it is not surprising that a high level of exercise empowerment may lead to a higher level of SPA.

In this study, digital media dependence, instability, and self-exploration were identified as antecedents directly related to exercise empowerment, which in turn was related to SPA. Specifically, higher levels of digital media dependence were associated with higher levels of exercise empowerment. In the future, mental health professionals should conduct baseline assessments, including quantifying levels of digital media dependence and exercise empowerment, prior to SPA interventions, which may aid in future treatment. Although instability was positively related to SPA, college students reporting higher levels of instability exhibited lower levels of exercise empowerment, which may help alleviate the impact of instability on social media dependence. This finding may be due to college students during unstable periods being more likely to prioritize career or work-related activities, such as spending a lot of time acquiring content knowledge (Bahk et al., 2010), theoretically spending less time acquiring exercise-related knowledge, and thus more likely to exhibit sedentary behavior, reducing their exercise empowerment (Strong et al., 2006). It is noteworthy that although self-exploration is negatively related to SPA, college students reporting higher levels of self-exploration exhibited higher levels of exercise empowerment, which may increase the impact of self-exploration on SPA. This finding may be due to college students with higher levels of self-exploration having higher expectations for themselves, with self-exploration seen as a motivator (Pienaar, 2022), and tending to take strategic actions (such as leaving a lasting impression on others) (Ranta et al., 2014), and thus engaging in more positive physical activities in order to achieve better external performance, resulting in higher levels of exercise empowerment, which may increase SPA levels. Therefore, providing work-related information and advertisements about the benefits of physical activity on campus seems necessary, but do not focus on the benefits of exercise solely on improving appearance, rather, promote a higher level of physical literacy and a positive perception of the benefits of exercise.

## 5 Limitations

The study has several limitations that should be considered when interpreting the results. Firstly, the cross-sectional study design used in this research prevents us from making definitive conclusions or assumptions about the longitudinal changes in the relationships between the evaluated variables and SPA. Secondly, the influence of gender on SPA may differ, but this aspect was not investigated in our study. Consequently, the primary factors affecting individual SPA may be dynamic, and the generalizability of our findings to other groups, such as emerging adults who are already in the workforce, may be limited. Therefore, future studies should explore whether these findings can be extended to other cohorts. Thirdly, previous research has shown that socioeconomic status, living situation (independence or living with parents), romantic relationship status, and parenting style are associated with specific characteristics of emerging adulthood, such as instability. Therefore, it is recommended that these variables be included in future research to gain a more comprehensive understanding of the relationship between EA characteristics and psychological health in college students. Lastly, the inclusion of participants within the lower age range of emerging adulthood in this study may restrict the generalizability of the findings to the broader emerging adult population.

## 6 Conclusion

It has been observed that specific characteristics of emerging adulthood, particularly instability and self-exploration, are indirectly related to SPA through their association with digital media dependence and exercise empowerment among college students. Therefore, our findings provide a new perspective and further evidence for improving SPA among college students in the emerging stage of adulthood. Interventions and strategies aimed at addressing these psychological factors may prove beneficial in reducing SPA among emerging adults, especially college students.

## Data availability statement

The raw data supporting the conclusions of this article will be made available by the authors, without undue reservation.

## Ethics statement

The studies involving humans were approved by the Ethics Committee of Guizhou Normal University. The studies were conducted in accordance with the local legislation and institutional requirements. The participants provided their written informed consent to participate in this study.

## Author contributions

LL was responsible for study design, data analysis, and manuscript writing. JY and YW were responsible for data collection. SB was responsible for literature review. All authors contributed to the article and approved the submitted version.
